# Predictors of improvement in disease activity in childhood and adolescent Crohn’s disease: an analysis of age, localization, initial severity and drug therapy — data from the Saxon Registry for Inflammatory Bowel Disease in Children in Germany (2000–2014)

**DOI:** 10.1007/s00431-024-05671-8

**Published:** 2024-08-03

**Authors:** Jens Weidner, Michele Zoch, Ivana Kern, Ines Reinecke, Franziska Bathelt, Ulf Manuwald, Yuan Peng, Elisa Henke, Ulrike Rothe, Joachim Kugler

**Affiliations:** 1 Medical Faculty Carl Gustav Carus, Institute for Medical Informatics and Biometry, TU Dresden, Fetscherstrasse 74, Dresden, 01307 Germany; 2 Medical Faculty Carl Gustav Carus Department of Health Sciences/Public Health, Institute and Policlinic for Occupational and Social Medicine, TU Dresden, Fetscherstrasse 74, Dresden, 01307 Germany; 3 Data Integration Center, Center for Medical Informatics, University Hospital Carl Gustav Carus, Fetscherstrasse 74, Dresden, 01307 Germany; 4 Thiem- Research GmbH, Carl-Thiem-Klinikum Thiemstr. 111, Cottbus, 03048 Germany; 5 University of Applied Sciences Dresden (FH-Dresden), Güntzstr. 1, Dresden, 01069 Germany; 6 GWT of TU Dresden, Dresden, Germany

**Keywords:** Crohn’s disease, IBD, Pediatrics, Therapy

## Abstract

The escalating worldwide prevalence of Crohn’s disease (CD) among children and adolescents, coupled with a trend toward earlier onset, presents significant challenges for healthcare systems. Moreover, the chronicity of this condition imposes substantial individual burdens. Consequently, the principal objective of CD treatment revolves around rapid inducing remission. This study scrutinizes the impact of age, gender, initial disease localization, and therapy on the duration to achieve disease activity amelioration. Data from the Saxon Pediatric IBD Registry in Germany were analyzed over a period of 15 years. In addition to descriptive methods, logistic and linear regression analyses were conducted to identify correlations. Furthermore, survival analyses and Cox regressions were utilized to identify factors influencing the time to improvement in disease activity. These effects were expressed as Hazard Ratios (HR) with 95% confidence intervals. Data on the clinical course of 338 children and adolescents with CD were available in the registry. The analyses showed a significant correlation between a young age of onset and the severity of disease activity. It was evident that treatment with anti-TNF (Infliximab) was associated with a more favorable prognosis in terms of the time required for improvement in disease activity. Similarly, favorable outcomes were observed with the combination therapies of infliximab with enteral nutrition therapy and Infliximab with immunosuppressants.

*Conclusion*: Our analysis of data from the Saxon Pediatric IBD Registry revealed that the timeframe for improvement of disease activity in pediatric Crohn’s disease is influenced by several factors. Specifically, patient age, treatment modality, and initial site of inflammation were found to be significant factors. The study provides important findings that underline the need for individualized treatment.

## Introduction

Crohn’s disease (CD), similar to Ulcerative Colitis (UC) and Indeterminate Colitis (CI), is part of the spectrum of chronic Inflammatory Bowel Disease (IBD). IBD are immune-mediated conditions that can affect individuals of all ages. While these diseases were considered rare in children and adolescents in the past, their incidence has increased worldwide since the beginning of the 21st century, particularly due to CD, although the incidence varies considerably depending on the geographical region [1, 2]. Furthermore, an unfavorable trend has been observed in terms of the age at diagnosis, showing a shift toward early childhood years [3–5]. The implications of the emergence of new-onset diseases during early childhood are associated with significant individual and societal disease burdens [6]. In these conditions, there is a pattern of recurring or persistent inflammatory activity, causing an elevated loss of nutrients despite increased nutritional needs [7]. The consequences of these illnesses can manifest in children and adolescents through symptoms of deficiencies, growth and developmental retardation, and psychosocial stressors. Elevated disease activity in childhood correlates with adverse changes in body size, weight, bone density, and delayed puberty [8–10]. Particularly, growth retardation influences the psychosocial development of the affected individuals, correlating with negative self-perception among children and adolescents, leading to adverse self-evaluation of body image [11]. Frequent absences from school and education due to recurring inflammatory activity further contribute to heightened psychosocial stress among affected children and adolescents [12].

In this context, the primary therapeutic goal for children and adolescents with CD is to achieve remission with complete mucosal healing (deep remission), maintain remission, and ensure age-appropriate development [13, 14]. The primary purpose of this thesis is to investigate various factors such as age, gender, initial localization and drug therapy on disease activity and to analyze the influence of these factors on the timing of improvement of disease activity in CD based on data from the Saxon Pediatric IBD Registry in Germany. The aim is to use these data to contribute to the understanding of the above-mentioned factors in relation to the temporal dynamics of disease improvement. The findings from this study may contribute to achieving the primary therapeutic goal of deep remission in pediatric Crohn’s disease.

## Methods

### Data source

The Saxon Pediatric IBD Registry in Germany has collected prospective, clinical and epidemiological data on children with IBD over a period of 15 years, from 2000 to 2014. This comprehensive registry operation took place within Saxony, a German federal state covering an area of 18,415 square kilometers and home to approximately 4.3 million residents [5, 15]. Exclusively included in the registry were individuals within the pediatric demographic residing in Saxony, diagnosed with IBD between January 1, 2000, and December 31, 2014, and who were below 18 years of age at the time of registry inclusion. All 31 pediatric hospitals in Saxony proactively provided data for the registry, ensuring the inclusion of all children and adolescents diagnosed and treated by specialized pediatric gastroenterologists within a pediatric hospital or clinic in Saxony since the year 2000. The treating hospital provided patient data as standardized forms to the leading institution, the Medical Faculty “Carl Gustav Carus” at the Technische Universität Dresden (TUD). Prior informed consent was obtained from the parents. Between 2017 and 2020, the data underwent comprehensive scrutiny to ensure comple-teness, meticulous validation, and evaluation by the Department of Health Sciences/Public Health at the TUD. For the present study, however, adolescents who had reached the age of 18 at each respective follow-up examination were excluded.

### Data preparation

The most recent confirmed diagnosis (according to the Porto criteria) [5, 16, 17] and the date of initial diagnosis were validated for calculations. IBD patients were defined as patients who met the endoscopic, histologic, diagnostic imaging, clinical and laboratory parameters according to the Lennard-Jones criteria [18], since 2005 according to the Porto criteria. [5, 16, 17]. To accomplish the aim of our investigation, we operationalized disease activity through the utilization of the Pediatric Crohn’s Disease Activity Index (PCDAI). This index comprises three main categories: 1st clinical symptoms, 2nd physical examination findings, and 3rd laboratory results. Typically, the score is scaled from 0 to 100. For the assessment of disease activity, the following cut-off values are applied: Remission <10, mild 10–30, moderate/severe 30–100 [19]. Improvement in disease activity was defined as falling below the respective cut-off value. This means an improvement in disease activity is given if the disease activity has changed from (1) moderate/severe to mild or remission or (2) from mild to remission. In order to investigate the influence of age, the ages of children and adolescents were dichotomized according to the “Paris classification” into A1a (0–10 years) and A1b (>10–17 years) [20]. Another variable used for the analysis is the localization of inflammation which was aggregated in accordance with the “Paris classification” [20]. In this context represents:

- L1 distal 1/3 ileum with ± limited cecal involvement
- L2 colon involvement
- L3 ileocolonic inflammation
- L4a an upper inflammation proximal to the ligament of Treitz
- L4b an upper inflammation distal to the ligament of Treitz and proximal to the distal 1/3 ileum

For the purposes of this study, L4a and L4b were consolidated into a single variable. To evaluate the duration until the improvement of disease activity, we calculated the variable “time” as the temporal difference between the admission date in the registry and the corresponding examination date.

Therapeutic categories include immunosuppressants (such as mercaptopurine and azathioprine), biologics (including infliximab and adalimumab) and steroids (such as budesonide, prednisone, dexamethasone and methylprednisolone). While the efficacy of mesalazine in the treatment of CD remains controversial and there is no evidence-based support for its role as induction therapy in CD, its use has been recommended in certain cases of very mild disease (Rümele, 2014). Therefore, we decided to include mesalazine treatment as a therapeutic category, which was supported by the registry data. In addition, categories were defined based on data and the consensus guidelines of the European Society for Paediatric Gastroenterology Hepatology and Nutrition (ESPGHAN) and the European Society for the Treatment of Pediatric Crohn’s Disease, which combine different treatment options.

### Statistical analysis

The statistical analyses were conducted in R Version 4.2.2. Beyond descriptive statistics, inferential statistical methods were applied for the analyses. Logistic and linear regression analyses were performed to elucidate associations, with the dependent variable consistently represented disease activity operationalized through the PCDAI score. The duration until improvement in disease activity was assessed through survival time analyses, wherein the transition from moderate/severe disease activity (PCDAI >30–100 points) to mild disease activity (PCDAI 10–30 points), and from mild disease activity to remission (PCDAI < 10 points), represented the defined event. The time to onset of improvement was determined utilizing the Kaplan-Meier estimator and was related to the time of inclusion in the registry (${t_0}$). Various inclusion times in the registry were synchronized with this predefined time. A Cox proportional hazard model was employed to calculate the probability of achieving improvement in disease activity, with time to improvement serving as the dependent variable. The effects were quantified as hazard ratios with 95% confidence intervals. A significance level of 5% was established for all statistical analyses. For the analyses, no patients were excluded from the dataset.

### Compliance with ethical standards

The registry design received approval from the Ethics Committee of the University of Leipzig (Reg. No. 033/2000). All procedures were conducted in compliance with ethical standards established by institutional and national research, as well as the Helsinki Declaration or equivalent ethical benchmarks. In the case of underage patients, written parental consent was obtained for the processing and storage of personal and medical data, as governed by the data protection vote. To uphold confidentiality, all patient data were pseudonymized, utilizing a unique identifier or patient number throughout the entire database. These data management practices adhered to the data protection regulations of the Federal Republic of Germany and Europe.

## Results

### Overall description of the registry population

Within the observational period spanning from 2000 to 2014, a cohort of 338 children and adolescents diagnosed with CD was enrolled in the registry and, consequently, deemed eligible for the current investigation. The dataset for these 338 individuals comprised a total of 3605 documentation forms from the registry, which have been incorporated into the analyses conducted for this study.

### Description of the registry population at the time of inclusion in the registry

In the registry, 214 (63.3%) males and 124 (36.7%) females were enrolled. The median age of children and adolescents diagnosed with CD at time point ${t_0}$ was 11.96 years (IQR: 4.16), with males averaging 11.90 years (IQR: 4.51) and females 11.90 years (IQR: 4.23). No significant difference in age between genders was observed (t-test: p-value = 0.315). Approximately one-fourth of the children (n=88, 26.0%) belonged to age group A1a (<10 years), while approximately three-fourths (250, 74.0%) were assigned to age group A1b (10–17 years).

**Table 1:** Disease activity at time point ${t_0}$ by gender N=326

	Remission		mild		moderate/severe	
male	68	20.86%	114	34.97%	24	7.36%
female	29	8.90%	75	23.01%	16	4.90%

**Fig. 1:**
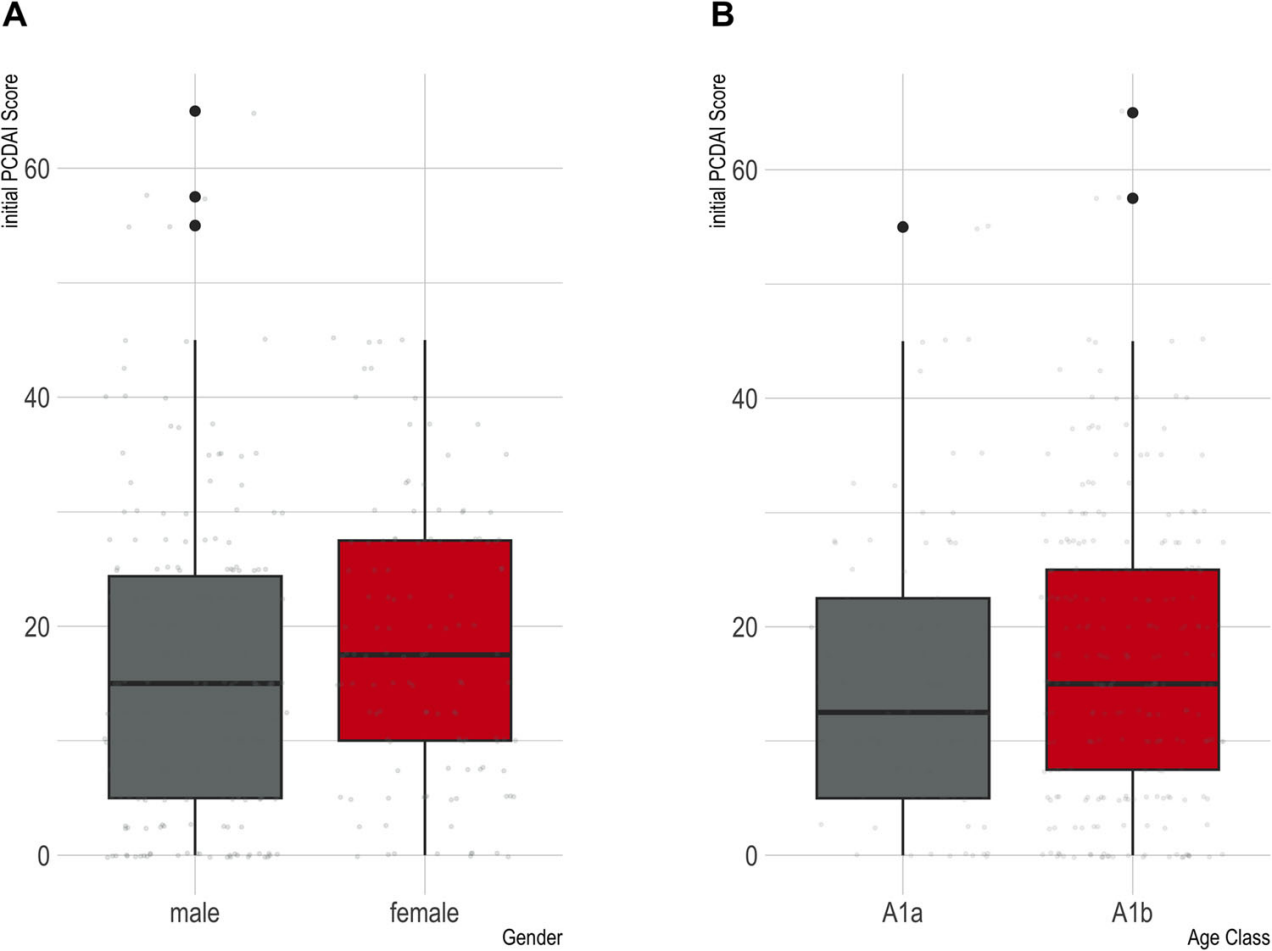
Initial disease activity with respect to: **A** Gender — There was no statistically significant difference in the initial disease activity between genders (t-test: p-value = 0.10), **B** Age group — No statistically significant difference in the initial disease activity was observed across distinct age groups (t-test: p-value = 0.39)

### Initial disease activity (PCDAI) at time point ${t_0}$

At time point $t_0$, an assessment of disease activity was available for 326 individuals, encompassing 95.8% of the pediatric and adolescent cohort. The average initial PCDAI score was 16.89 points (SD 12.81), with a median score of 15.0, spanning from 0 to 65 on a scale of 0 to 100. Table 1 shows the distribution of disease activity by gender at the time of inclusion in the registry (${t_0}$).

**Table 2:** Distribution of disease activity by age group

	Disease activity		
Age group	Remission	mild	moderate/severe
A1a (%)	27 (30.7)	47 (53.40)	10 (11.36)
A1b (%)	142 (56.8)	30 (12.0)	69 (27.6)

At the time of inclusion in the registry ($t_0$), the mean PCDAI score for individuals in age group A1a was 15.85 points (SD 13.11), while those in age group A1b exhibited a mean score of 17.26 points (SD 12.72). Analyzing the data by gender, the mean PCDAI score at ${t_0}$ for males was 16.0 points (SD 13.22), whereas for females, it was 18.42 points (SD 17.59). No statistically significant disparity in initial disease activity was observed for both age groups and genders, as presented in Fig. 1. As shown in Table 2, approximately 30.7% of the children in age group A1a and 56.8% of the children and adolescents in age group A1b showed a PCDAI (Pediatric Crohn’s Disease Activity Index) score of < 10 points, indicating remission. At time $t_0$, mild disease activity was observed in 53.4% of children in age group A1a and in 12.0% of children in age group A1b. In addition, at time $t_0$, 11.36% of the children in age group A1a and 27.6% of the children in age group A1b had moderate to severe disease activity (Table 2).

In a subsequent analytical phase, an investigation was undertaken to elucidate the potential association between inflammation localization at time point ${t_0}$ and disease activity. To explore this relationship, a binary logistic regression analysis was executed. No significant correlations were identified between the specific localization and the PCDAI score. The results of this regression analysis are shown in Table 3.

**Table 3:** Results binary logistic regression analysis; dependent variable PCDAI, independent variable localization

Localization	Estimate	p-value	Odds Ratio	95% CI
L1	$-$0.232	0.61	0.79	0.31–1.95
L2	$-$4.498	0.24	0.61	0.26–1.41
L3	0.225	0.54	1.25	0.43–3.65
L4	0.737	0.08	2.09	0.90–4.94

**Fig. 2:**
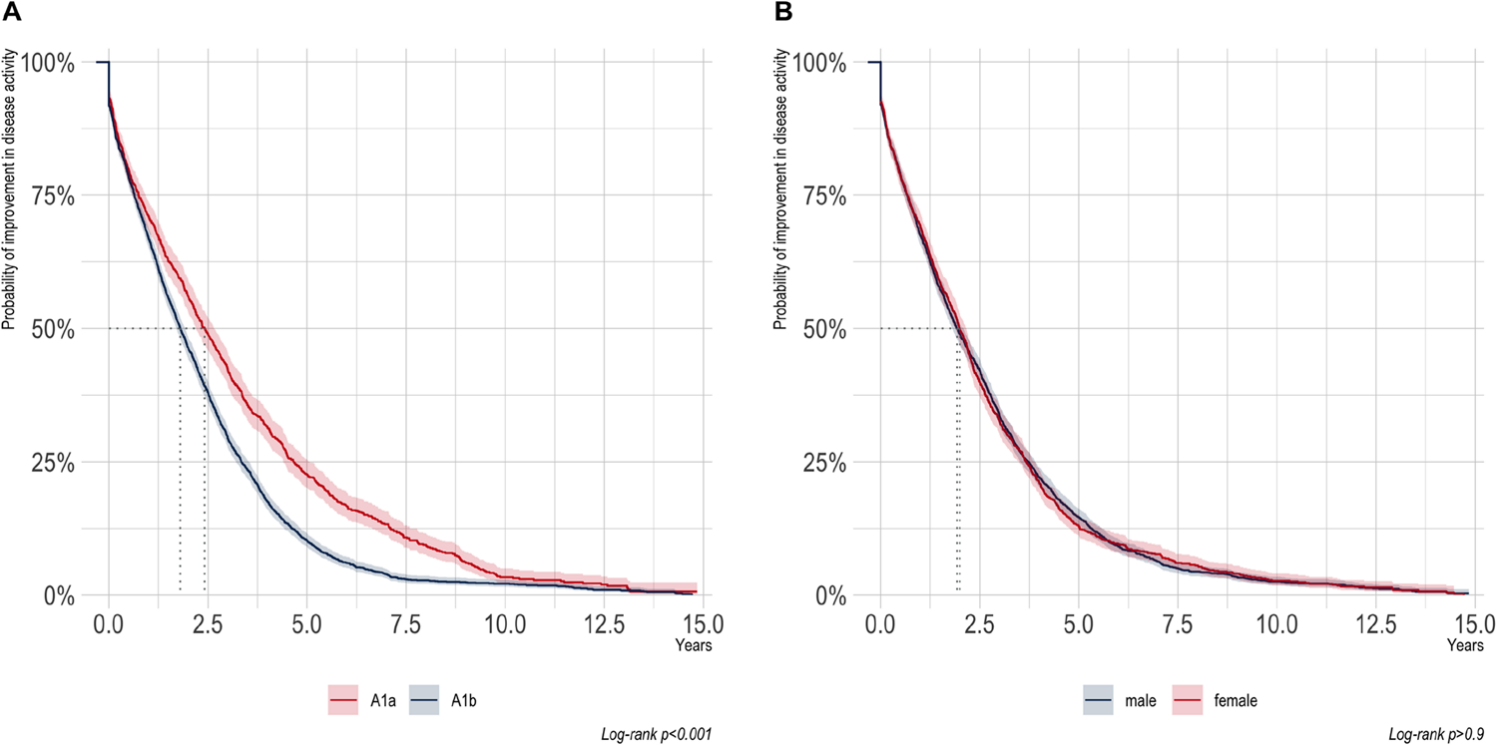
Survival time analysis event= improvement in disease activity. **A** Stratified by age group A1a median time to improvement 2.41 years (95% CI: 2.19–2.65); A1b 1.80 years (95% CI: 1.70–1.92) log-rank test p<0.001. **B** Stratified by gender boys median time to improvement 1.93 years (95% CI: 1.81–2.09); girls 2.00 years (95% CI: 1.88–2.16), log-rank test: p= 0.3

**Table 4:** Cox proportional hazard model: independent variables: initial localization (L1–L4) age classes (A1a, A1b) and gender, Signif. codes: “$$^{***}$$” 0.001, “$$^{**}$$” 0.01, “$$^{*}$$” 0.05

Predictor	coef	p-value	exp(Coef)	exp(-Coef)	95% CI [exp(Coef)]
L1 (Reference = 0)	$-$0.04	0.570	0.96	1.04	0.83–1.11
L2 (Reference = 0)	$-$0.03	0.690	0.97	1.03	0.83–1.12
L3 (Reference = 0)	$-$0.13	0.170	0.88	1.14	0.73–1.06
L4 (Reference = 0)	0.61	0.000	1.88$$^{***}$$	0.54	1.33–1.54
Age Group: A1b (Reference = A1a)	0.32	0.000	1.37$$^{***}$$	0.72	1.26–1.49
Gender: female (Reference = male)	0.02	0.63	1.02	0.98	0.94–1.01

### Age, gender and localization as influencing factors on the duration until improvement of disease activity

In a subsequent analysis, we directed our focus toward the temporal dynamics of improvement in disease activity. The median time to achieve an improvement in disease activity in the overall population was determined to be 1.95 years (95% CI 1.87–2.07), with the median time to the subsequent follow-up visit being 1.77 years. In addition, the influence of age, represented by the different age groups A1a and A1b, as well as the influence of gender on the time to improvement in disease activity were investigated. As shown in Fig. 2A, the median time to improvement in disease activity in age group A1b was significantly shorter than in age group A1a (A1a: 2.41 years vs. A1b: 1.80 years) (Fig. 2A). The median time to the next follow-up visit was 2.18 years for age group A1a and 1.63 years for age group A1b. The median time to improvement in disease activity was 1.93 years for boys and 2.00 years for girls. No significant difference in the duration until improvement induction could be identified (Fig. 2B). A Cox regression was also performed. The result showed that the hazard ratio for achieving improvement in disease activity was significantly 39% higher in age group A1b (HR 1.43; 95% CI: 1.29–1.51, p-value< 0.0001) than in age group A1a.

Regarding follow-up time, it was observed that the median follow-up times for disease activity varied (remission: 1.98 years, mild: 1.44 years, moderate/severe: 1.51 years). The differences in follow-up times were significant in the ANOVA performed (F-test p< 0.001), with the post-hoc test indicating a significant difference only between remission and mild (Tukey-HSD p < 0.001). The follow-up times did not correlate with the timing of disease improvement.

**Fig. 3:**
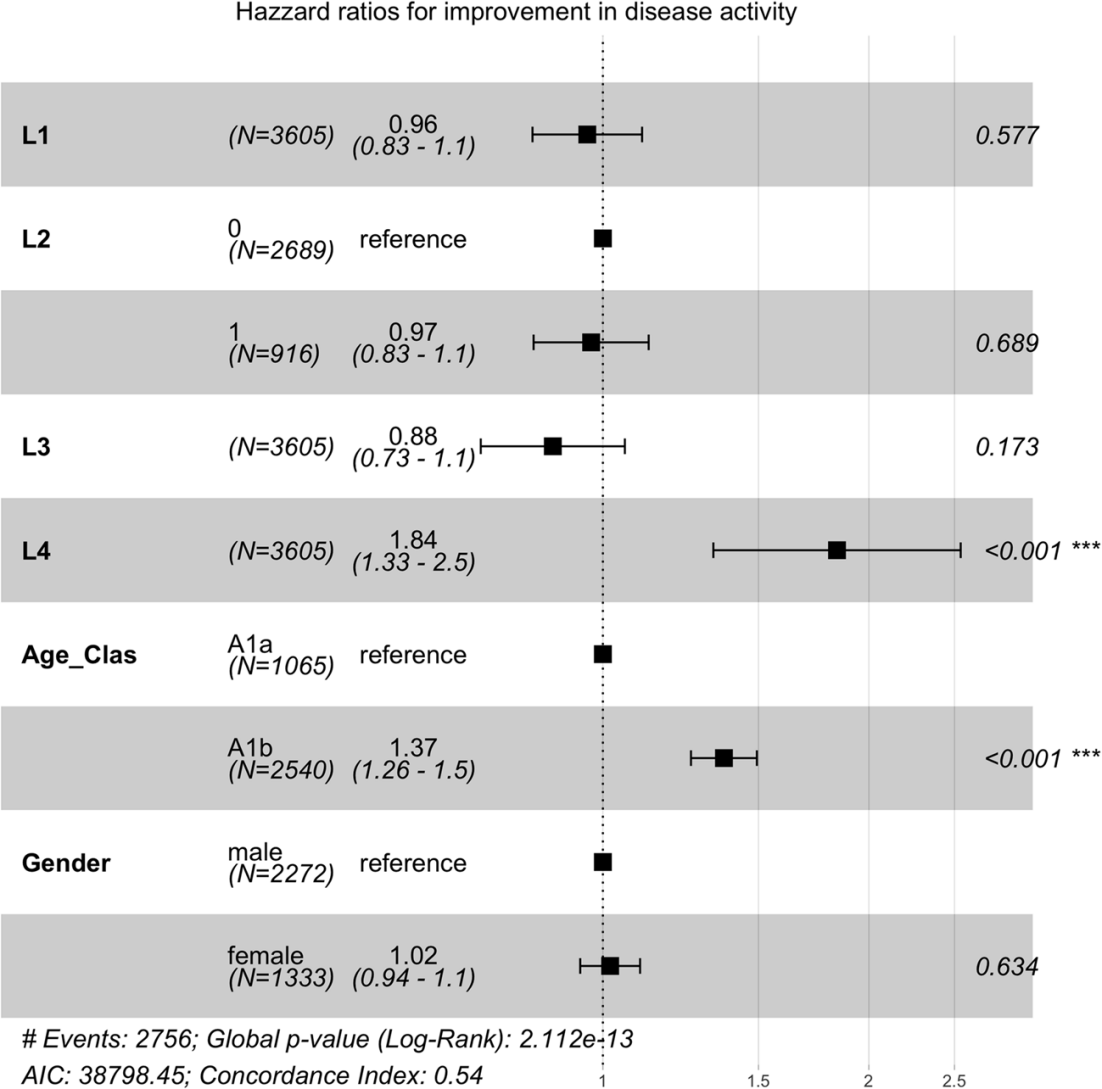
Cox proportional hazard model reduced model: independent variable localization, age classes and gender

In a further step, the original localization of the inflammation was included in the Cox model as a further possible predictor alongside age. It was found that disease localization limited to L4 is associated with a more favorable prognosis in terms of improvement in disease activity (Table 4, Fig. 3). However, it should be noted that this result is moderated by the age groups.

### Therapy as an influencing factor on the duration until improvement in disease activity

#### Drug monotherapy

At time $t_0$, 38.63% of children in age group A1a were administered mesalazine, while 44.8% in age group A1b received the same. Steroids were prescribed to 51.1% in age group A1a and 42.0% in A1b. Immunomodulatory therapy was initiated in 35.2% of subjects in group A1a and 41.2% in A1b at time $t_0$. Infliximab treatment was administered to 13.6% of children in age group A1a and 7.6% in A1b. Nutritional therapy was provided to approximately 12.5% of children and adolescents in both age groups at time $t_0$ (Table 5). Table 5 also presents the distribution of prescribed therapeutic combinations between the age groups.

**Table 5:** Distribution of treatment options between age groups A1a and A1b

Treatment	A1a (%)	A1b (%)
Mesalazine	34 (38.63)	112 (44.80)
Steroids	45 (51.13)	105 ( 42.0)
Immunosuppressants	31 (35.22)	103 ( 41.20)
Infliximab	12 (13.63)	19 (7.60)
Nutrition	11 (12.50)	31 (12.40)
Nutrition and Mesalazine	6 (6.81)	17 (6.80
Steroids and Immunosupressants	15 (17.04)	58 (23.2)
Infliximab and Nutrion	3 (3.40)	2 (0.8)
Nutrtion and Steroids	7 (7.95)	17 (6.80)
Immunosupressants and Infliximab	3 (3.40)	9 (3.60)

In the subsequent analysis, the duration until the onset of an improvement in disease activity was examined, taking into account the specific drug therapies. First, the prescribed monotherapies were analyzed. Across the age groups A1a and A1b, the drug treatment of children and adolescents with infliximab showed the shortest median time until an improvement in disease activity was achieved (Table 6). In terms of age groups, the results show that children and adolescents in age group A1b benefit earlier from the prescribed monotherapies compared to age group A1a, as shown in Table 6 and Fig. 6 in Appendix A. Only in connection with infliximab therapy did the A1a age group show advantages over the A1b age group in the survival time analysis. At time $t_0$, 9% (n=31) of the children and adolescents had previously received treatment with infliximab. Among these individuals, 19 exhibited mild disease activity, while 2 demonstrated moderate to severe disease activity. Intriguingly, 10 children were in remission at this time point. However, these differences did not reach statistical significance in the log-rank test (Table 7, Fig. 6 in Appendix A).

**Table 6:** Survival analysis: influence of each of the five monotherapies on the duration until improvement in disease activity

Treatment with:	n	events	median time	95% CI
Mesalazine	1752	1576	2.36	2.23–2.51
Steroids	1828	1639	2.20	2.05–2.33
Immunosuppressants	1752	1581	2.10	1.96–2.21
Infliximab	515	463	1.75	1.49–1.96
Nutrition	344	299	2.13	1.70–2.58

**Table 7:** Survival analysis: influence of medication (monotherapy) on the duration until improvement in disease activity by age group

Treatment with	n	events	median time	95% CI	n	events	median time	95% CI	Log-rank
									p-value
	Age Group A1a				Age Group A1b				
Mesalazine	540	470	2.97	2.62–3.24	1212	1106	2.17	1.98–2.33	<0.001
Steroids	584	514	2.48	2.22–2.76	1244	1125	2.09	1.93–2.23	<0.001
Immunosuppressants	554	485	2.45	2.20–2.82	1198	1096	1.94	1.80–2.11	<0.001
Infliximab (TNF$\alpha$)	183	166	1.63	1.27–2.06	332	297	1.78	1.58–2.09	0.2
Nutrition therapy	105	90	2.53	1.86–3.96	239	209	1.90	1.37–2.51	0.002

**Fig. 4:**
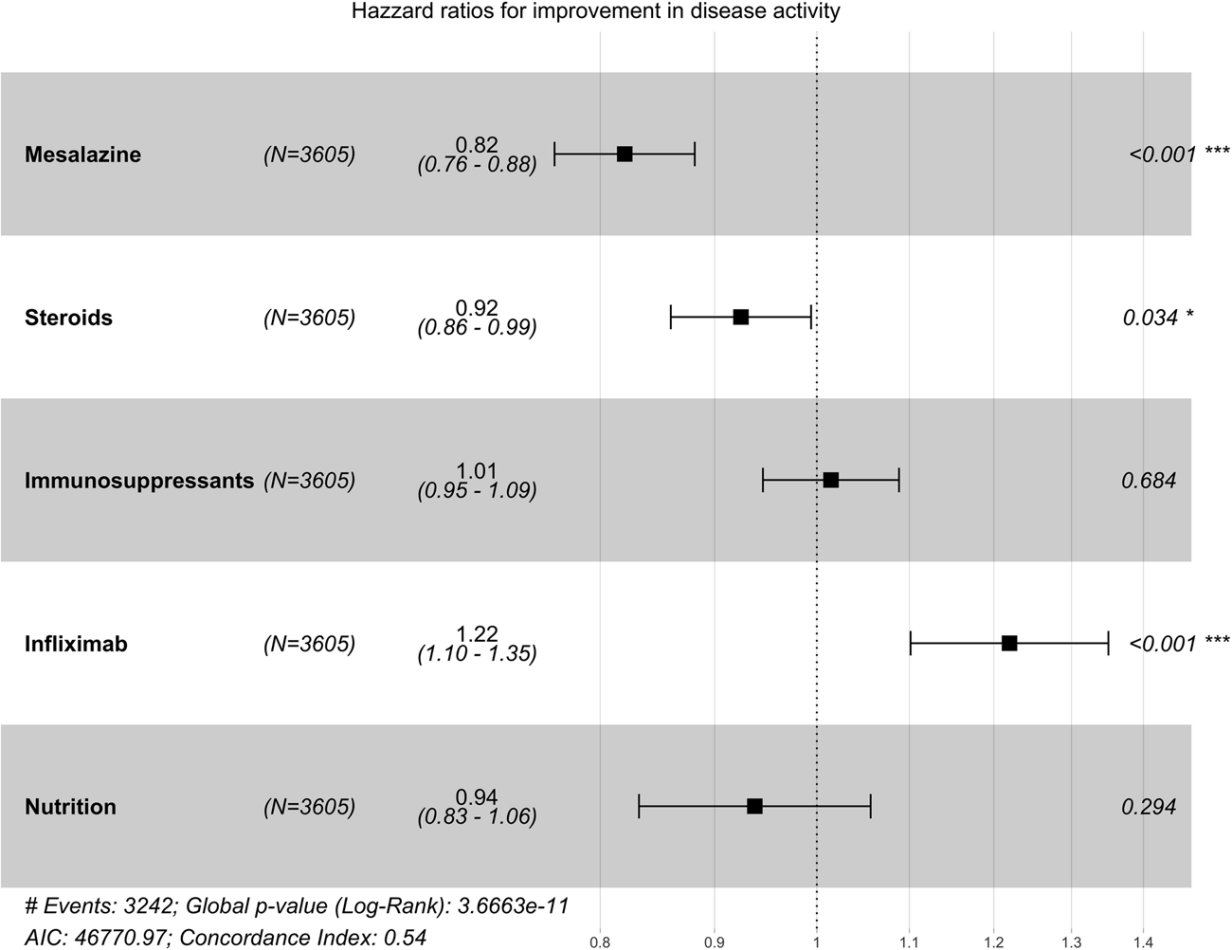
Cox proportional hazard model: independent variable medication (monotherapy)

A Cox regression was then carried out in a further step to investigate the influence of the respective drug monotherapy on the duration until the onset of an improvement in disease activity. Globally, a significant log-rank test (p-value <0.001) was found, which indicates a heterogeneous effect of the drug monotherapies on the duration until improvement in disease activity. In the Cox proportional hazard model, infliximab monotherapy was associated with a more favorable prognosis in terms of duration to onset of improvement in disease activity (HR: 1.22, 95% CI: 1.10–1.35). Figure 4 also shows the superiority of infliximab therapy over other drug monotherapies across the two age groups. Interestingly, therapy with mesalazine (HR: 0.82, 95% CI: 0.76–0.88) and with steroids (HR: 0.92, 95% CI: 0.86–0.99) was not associated with a positive effect on the duration until the onset of improvement in disease activity. Similarly, nutritional therapy alone (HR: 0.94, 95% CI: 0.83–1.06) was not associated with a positive effect on the duration to improvement of disease activity in the registry population; however, this result was not statistically significant (Fig. 4).

Infliximab is associated with a shorter time to achieve improvement in disease activity compared to alternative monotherapies, as evidenced by the age group-specific analysis. Here, age group A1a with an HR of 1.48 (95% CI: 1.24–1.76) appears to be superior to age group A1b with an HR of 1.12 (0.99–1.27) (Fig. 7 in Appendix A). However, the advantage of infliximab in age group A1b compared to other drug monotherapies was not significant (see Fig. 7 in Appendix A).

**Table 8:** Survival analysis: influence of combination treatment on the duration until improvement in disease activity in all age groups

Combination Treatment with:	n	events	median time	95% CI
Nutritional therapy and mesalazine	181	160	1.88	1.50–2.62
Steroids and immunosuppressants	1002	900	2.13	1.96–2.3
Infliximab and nutritional therapy	58	51	1.95	1.20–2.62
Nutritional therapy and steroids	209	179	2.10	1.56–2.58
Immunosuppressants and Infliximab	247	225	1.98	1.75–2.27

**Fig. 5:**
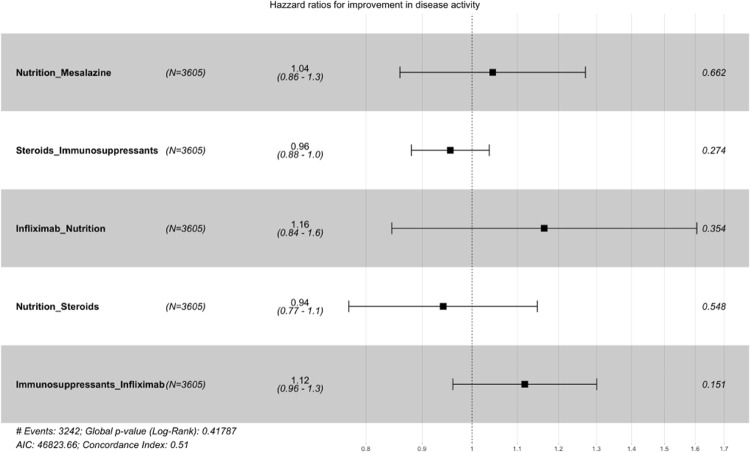
Cox proportional hazard model: independent variable medication (combination therapy)

#### Drug combination therapy

In order to also examine the impact of treatment combinations, we replicated the earlier analyses, this time incorporating the treatment combinations as independent variables. Table 8 reveals that combination treatments involving nutritional therapy and mesalazine (HR: 1.88, 95% CI: 1.50–2.62), infliximab and nutritional therapy (HR: 1.95, 95% CI: 1.20–2.62), and a combination therapy of immunosuppressants and infliximab (HR: 1.98, 95% CI: 1.75–2.27) are linked to a shorter median time for the induction of improvement in disease activity. However, the somewhat wide confidence intervals suggest that these estimates lack robustness, likely due to a limited sample size. This presumption was subsequently corroborated by Cox regression analysis, which failed to identify any statistically significant effects regarding the duration until the induction of improvement in disease activity (Fig. 5). However, Fig. 5 indicates a tendency in favor of a combination treatment involving infliximab and nutritional therapy, or a combination therapy with immunosuppressants and infliximab.

**Fig. 8:**
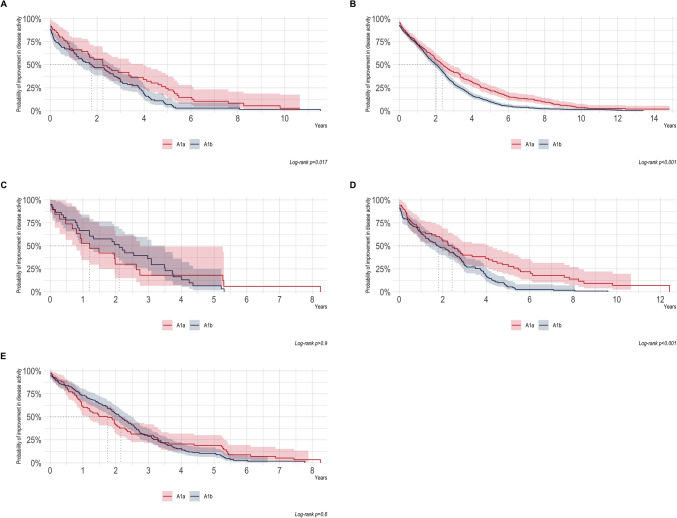
Duration until improvement in disease activity depending on combination therapy and age groups. **A** Kaplan-Meier plot for Combination treatment with nutritional therapy and mesalazine (see data in Table 6). **B** Kaplan-Meier plot for Combination treatment with steroids and immunosuppressants (see data in Table 6). **C** Kaplan-Meier plot for Combination treatment with infliximab and nutritional therapy (see data in Table 6). **D** Kaplan-Meier plot for Combination treatment with nutritional therapy and steroids (see data in Table 6). **E** Kaplan-Meier plot for Combination treatment with immunosuppressants and infliximab (see data in Table 6)

In the age group-specific analysis, combinations of treatment involving nutritional therapy and mesalazine, as well as nutritional therapy and steroids, exhibited a significantly shorter median time for inducing improvement in disease activity in age group A1b compared to age group A1a (Table 9, Fig. 8 in Appendix B). Conversely, for age group A1a, the combinations of infliximab treatment with nutritional therapy, along with the combination of immunosuppressants and infliximab, appeared to confer advantages in terms of the duration until the induction of an improvement in disease activity. However, these findings did not reach statistical significance in the log-rank test between the age groups. It is worth noting that some results are characterized by wide confidence intervals, particularly in groups with smaller case numbers (Table 9, Fig. 8 in Appendix B).

**Table 9:** Survival analysis: influence of combination treatments on the duration until improvement in disease activity by age group

Combination treatment	n	events	median time	95% CI	n	events	median time	95% CI	Log-rank
with:									p-value
	Age Group A1a				Age Group A1b				
Nutritional therapy and	61	53	2.24	1.63–3.96	120	107	1.76	1.21–2.68	0.01
mesalazine									
Steroids and	311	269	2.36	2.01–2.78	691	631	2.02	1.81–2.23	<0.001
immunosuppressants									
Infliximab and	20	18	1.2	0.80–2.73	38	33	2.10	1.20–3.49	>0.9
nutritional therapy									
Nutritional therapy and	69	56	2.43	1.69–4.11	140	123	1.79	1.33–2.58	<0.001
steroids									
Immunosuppressants	83	75	1.75	1.15–2.12	164	150	2.15	1.88–2.48	0.6
and infliximab									

**Fig. 9:**
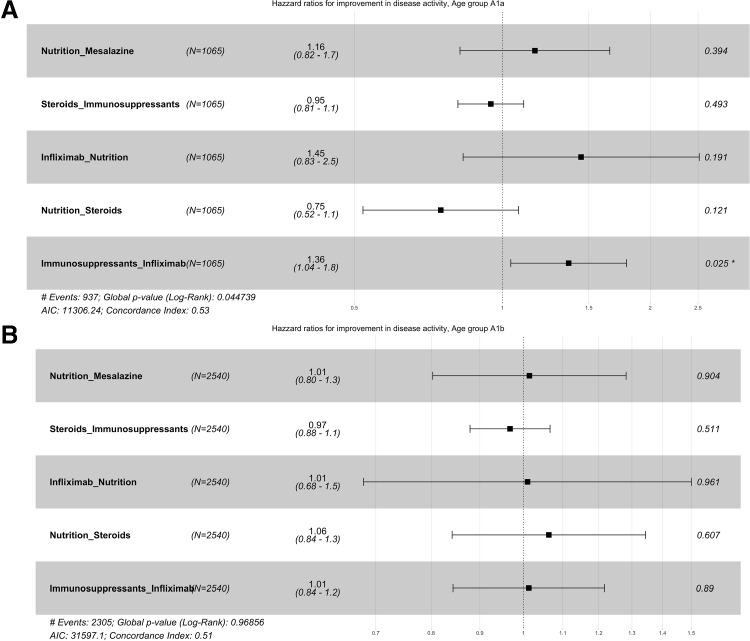
Cox proportional hazard model age group specific: independent variable medication (combination therapy). **A** Cox proportional hazard model age group A1a. **B** Cox proportional hazard model age group A1b

In the subsequent Cox regression analysis (Fig. 9 in Appendix B), within age group A1a, the combination treatment involving immunosuppressants and infliximab was notably linked to a significantly higher likelihood of a shorter time to the induction of improvement in disease activity. While combinations such as nutritional therapy with mesalazine and infliximab with nutritional therapy exhibited positive trends in terms of the duration of disease improvement, these effects did not reach statistical significance (Fig. 9A in Appendix B). Notably, for age group A1b, the Cox regression did not reveal any statistically significant effects. However, the presence of very wide confidence intervals was observed, likely attributable to the limited sample size (Fig. 9B in Appendix B).

## Discussion

In our analysis, we investigated the time duration until the induction of an improvement in disease activity in the context of pediatric CD. This paper focused on the influences of age, gender, inflammation localization and therapy. This study utilized data from the Saxon Pediatric IBD Registry in Germany and covered a time horizon of 15 years.

The rather long median times to an improvement in disease activity were particularly striking. This is attributed to the fact that the children and adolescents included in the registry had different intervals of up to 3 years between documented re-presentations, which is due to the methodology of the registry. To address this problem in the future, observational studies based on real-world data (RWD) would be useful. Conducting studies on large-scale data within the Observational Health Data Sciences and Informatics (OHDSI) community based on the Observational Medical Outcomes Partnership (OMOP) Common Data Model (CDM) [21–23] could help to investigate the topic further. Nevertheless, several interesting results were reported, which need to be interpreted and discussed in the light of current evidence.

Firstly, our findings revealed a correlation between the age of children and adolescents with CD and the duration of improvement in disease activity. According to the results, individuals in the A1b age group, with a median duration of 1.8 years, exhibited an advantage over those in the A1a age group. Simultaneously, the results suggest a more severe disease course for individuals in the A1a age group compared to their counterparts in the A1b age group. These outcomes align with existing evidence indicating that a younger age is associated with a more severe disease course [24–26]. However, it is pertinent to consider age-related variations in disease activity and antimicrobial seroreactivity in connection with the diagnosis. Our findings revealed that age group A1b exhibited higher PCDAI scores at time $t_0$ compared to age group A1a, although the median time to improvement in disease activity was shorter in A1b. Haberman et al. (2019) demonstrated heightened activity of specific immune genes, such as GM-CSF and INF$\gamma$, in older children and adolescents (A1b) relative to younger patients (A1a). Concurrently, they observed a reduction in the activity of antimicrobial genes in Paneth cells, notably $\alpha$-defensins, among older patients. This decline in $\alpha$-defensin expression correlated with an increase in INF$\gamma$ expression, which, in turn, was associated with heightened inflammatory activity. Notably, the A1b age group also exhibited a more better immune response [27].

In the context of the initial inflammation localization, analyses revealed that an initially limited localization to L4 was associated with a favorable prognosis concerning the duration until improvement in disease activity, albeit with a wide confidence interval accompanying this result. Therefore, this outcome should also be interpreted in light of the broad confidence interval. Secondly, our results showed different effects depending on the therapy with regard to the duration of improvement in disease activity. Monotherapy with infliximab is associated with a more favorable prognosis in the data evaluated in this study, with children in the A1a age group apparently having a better prognosis than those in the A1b age group. Treatment with anti-TNF drugs such as infliximab are considered highly effective drug therapies to induce both clinical improvement in disease activity and remission. The RISK study showed that early anti-TNF therapy had higher steroid- and surgery-free remission rates after 1 year than induction with dietary therapy or steroid treatment [28, 29]. Similarly, the results of the RISK study suggest that early treatment with Infliximab is associated with a significantly lower risk of developing penetrating B3 complications [30]. Primary therapy with anti-TNF alpha is recommended in the presence of risk factors for poor progression, such as young age [24, 28]. The results also showed that exclusive nutritional therapy is associated with a shorter time to improvement in disease activity compared to steroid or mesalazine therapy. Our results are supported by van Rheenen et al. [28]. In the context of the updated ECCO-ESPGHAN guideline on the medical treatment of pediatric CD, the benefit of enteral nutrition therapy was again confirmed in several meta-analyses [31–33]), so that the recommendation for induction therapy via exclusive enteral nutrition remains valid [24, 28]. In the context of combination therapy, combinations of infliximab treatment and nutritional therapy were shown to be beneficial compared to other therapies, with children in the A1a age group appearing to have the greater advantage. Similarly, the combinations of immunosuppressive therapy and treatment with infliximab were associated with a shorter time to induction of improvement in disease activity. For the A1b age group, a combination of nutritional therapy and the administration of mesalazine appeared to be beneficial. However, the effects were not significant in the Cox regression and the wide confidence intervals suggest imprecision in the estimates due to the small sample size. Thirdly, it can be concluded from our results that the treatment of pediatric CD should be individualized and tailored to the respective children and adolescents. Such an individual treatment plan should be drawn up taking into account age, disease location, disease behavior, growth retardation, side effects of medication and quality of life as well as in an interdisciplinary team consisting of pediatric gastroenterologists, social medicine, psychology and nursing. We thus follow the ECCO-ESPGHAN guidelines for the medical treatment of pediatric CD [24, 28].

**Limitations** Our study faces limitations stemming from the small sample sizes in the subgroup analysis. Furthermore, our analysis of the data from the Saxon Pediatric IBD Registry is limited by the specified methodology of the registry, which is associated with sometimes long intervals in the follow-up examinations of the children and adolescents.

## Conclusion

Our analysis of data from the Saxon Pediatric IBD Registry showed that the duration until improvement of disease activity in pediatric Crohn’s disease depends on various factors. In particular, the age of the patients, the type of therapy and the initial localization of inflammation play a role. Despite the methodological peculiarities of the registry, the analysis provided important findings that emphasize the need for individualized treatment, which should be developed in an interdisciplinary team taking into account age, disease characteristics and quality of life.

Future studies based on RWD and a CDM, such as the OMOP CDM, would be beneficial for precision of analyses. The OMOP CDM most closely matches the criteria for facilitating data sharing in longitudinal studies [23, 34]. This allows a more comprehensive and comparable collection of data on pediatric IBD and allows distributed data analysis across different sites. This could improve the understanding of CD and promote evidence-based strategies for prevention and intervention.

## Figures and Tables

**Fig. 6:**
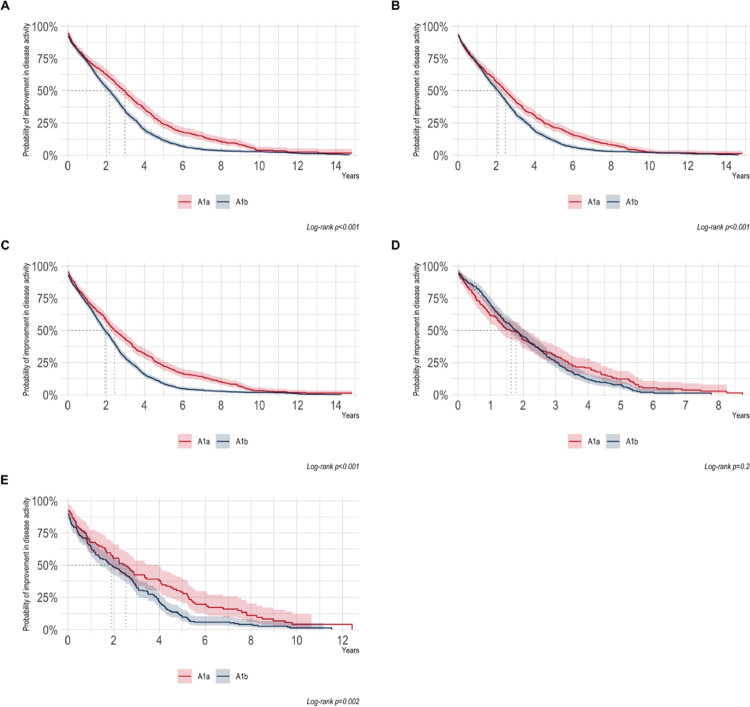
Duration until improvement in disease activity depending on drug monotherapy and age groups. **A** Kaplan-Meier plot for treatment with mesalazine (see data in Table 5). **B** Kaplan-Meier plot for treatment with Steroids (see data in Table 5). **C** Kaplan-Meier plot for treatment with Immunosuppressants (see data in Table 5). **D** Kaplan-Meier plot for treatment with Infliximab (see data in Table 5). **E** Kaplan-Meier plot for treatment with nutrition therapy (see data in Table 5)

**Fig. 7:**
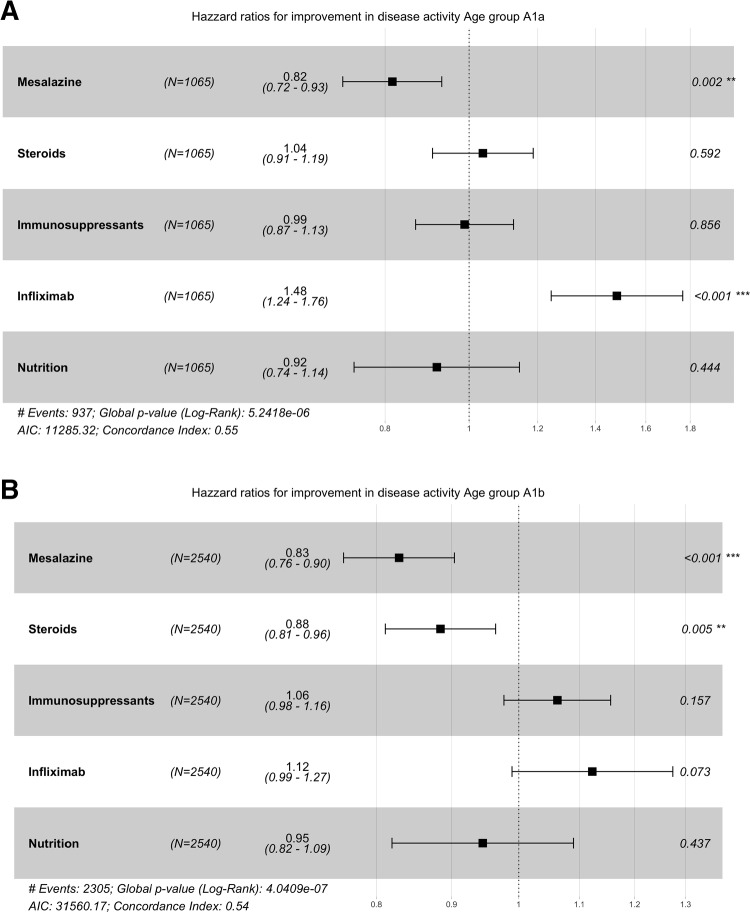
Cox proportional hazard model age group specific: independent variable medication (monotherapy). **A** Cox proportional hazard model age group A1a. **B** Cox proportional hazard model age group A1b

## Data Availability

The data sets generated and/or analyzed during this study are available from the corresponding author upon reasonable request.

## Abbreviations

**CD**: Crohn’s disease
**CDM**: Common Data Model
**EET**: exclusive enteral nutrition therapy
**IBD**: Inflammatory bowel disease
**CI**: Colitis indeterminata
**OHDSI**: Observational Health Data Sciences and Informatics
**OMOP**: Observational Medical Outcomes Partnership
**PCDAI**: Pediatric Crohn’s Disease Activity Index
**PET**: Partial Enteral nutrition Therapy
**UC**: Ulcerative colitis

## Declarations

### Statement on the use of generative AI

This manuscript was written and finalized by the authors without the help of an AI such as ChatGPT or other generative language models.
